# Atopic diseases and inflammation of the brain in the pathogenesis of autism spectrum disorders

**DOI:** 10.1038/tp.2016.77

**Published:** 2016-06-28

**Authors:** T C Theoharides, I Tsilioni, A B Patel, R Doyle

**Affiliations:** 1 Molecular Immunopharmacology and Drug Discovery Laboratory, Department of Integrative Physiology and Pathobiology, Tufts University School of Medicine, Boston, MA, USA; 2 Sackler School of Graduate Biomedical Sciences, Program in Cell, Molecular and Developmental Biology, Tufts University, Boston, MA, USA; 3 Department of Internal Medicine, Tufts University School of Medicine and Tufts Medical Center, Boston, MA, USA; 4 Department of Psychiatry, Tufts University School of Medicine and Tufts Medical Center, Boston, MA, USA; 5 Department of Child Psychiatry, Harvard Medical School, Massachusetts General Hospital and McLean Hospital, Boston, MA, USA

## Abstract

Autism spectrum disorders (ASDs) affect as many as 1 in 45 children and are characterized by deficits in sociability and communication, as well as stereotypic movements. Many children also show severe anxiety. The lack of distinct pathogenesis and reliable biomarkers hampers the development of effective treatments. As a result, most children with ASD are prescribed psychopharmacologic agents that do not address the core symptoms of ASD. Autoantibodies against brain epitopes in mothers of children with ASD and many such children strongly correlate with allergic symptoms and indicate an aberrant immune response, as well as disruption of the blood–brain barrier (BBB). Recent epidemiological studies have shown a strong statistical correlation between risk for ASD and either maternal or infantile atopic diseases, such as asthma, eczema, food allergies and food intolerance, all of which involve activation of mast cells (MCs). These unique tissue immune cells are located perivascularly in all tissues, including the thalamus and hypothalamus, which regulate emotions. MC-derived inflammatory and vasoactive mediators increase BBB permeability. Expression of the inflammatory molecules interleukin (IL-1β), IL-6, 1 L-17 and tumor necrosis factor (TNF) is increased in the brain, cerebrospinal fluid and serum of some patients with ASD, while NF-kB is activated in brain samples and stimulated peripheral blood immune cells of other patients; however, these molecules are not specific. Instead the peptide neurotensin is uniquely elevated in the serum of children with ASD, as is corticotropin-releasing hormone, secreted from the hypothalamus under stress. Both peptides trigger MC to release IL-6 and TNF, which in turn, stimulate microglia proliferation and activation, leading to disruption of neuronal connectivity. MC-derived IL-6 and TGFβ induce maturation of Th17 cells and MCs also secrete IL-17, which is increased in ASD. Serum IL-6 and TNF may define an ASD subgroup that benefits most from treatment with the natural flavonoid luteolin. Atopic diseases may create a phenotype susceptible to ASD and formulations targeting focal inflammation of the brain could have great promise in the treatment of ASD.

## Introduction

Autism spectrum disorders (ASDs) are pervasive neurodevelopmental disorders characterized by deficits in communication and social interactions, as well as the presence of stereotypic behaviors.^1, 2, 3^ Numerous gene mutations have been identified in patients with ASD, but no direct link has so far been uncovered except for a small percentage of cases associated with Tuberous Sclerosis, Fragile X syndrome, Rett syndrome and *PTEN* deficiency.^4, 5^ As a result, even though there are a number of genetically-engineered mice with phenotypes resembling autism,^6^ they do not adequately reflect ASD and there is an urgent need for appropriate animal ‘models' of ASD.^7^ In fact, mouse ‘models' are increasingly considered unreliable with respect to inflammatory human diseases.^8^ We recently reported that a small number of bull terriers develop symptoms consistent with autism and have increased serum neurotensin (NT) and corticotropin-releasing hormone (CRH), also found to be elevated in children with ASD.^9^

ASD may affect as many as 1 in 45 children in the USA,^10^ but the global prevalence is still under-recognized.^11^ The lack of reliable biomarkers^12^ and specific pathogenesis,^13^ as well as the existence of subgroups or comorbidities^14^ (Table 1), makes the development of specific treatments and conducting clinical studies difficult.^13^ As a result, child and adolescent outpatient mental health services in the USA have increased considerably.^15^ Moreover, the annual economic burden for ASD was recently estimated at $268 billion for 2015 and is projected to reach $416 billion in 2025.^16^

A number of perinatal allergic, genetic, environmental, immune and infectious factors may increase the risk of or contribute to the pathogenesis of ASD^17, 18, 19^ (Table 2). These could act through activation of a unique tissue immune cell, the mast cell (MC).^20, 21^ MCs derive from bone marrow progenitors and mature in tissues depending on microenvironmental conditions.^22^ In addition to histamine, stimulated MCs secrete other vasoactive and pro-inflammatory mediators such as the preformed kinins and proteases, as well as the *de novo* synthesized leukotrienes, prostaglandins, chemokines (CCXL8, CCL2), cytokines (interleukin (IL)-4, IL-6, IL-1, tumor necrosis factor (TNF)) and vascular endothelial growth factor (VEGF).^20^

MCs are not only considered critical for the development of allergic reactions,^20^ but also for immunity^22^ and inflammation.^23^ In fact, many studies have reported that allergic diseases in preschoolers are strongly associated with psychological and behavioral problems.^24^ We had proposed that MC-derived mediators could disrupt the blood–brain barrier (BBB) and cause 'allergy of the brain'^25^ or ‘focal encephalitis',^26^ thus contributing to the pathogenesis of ASD.^26, 27^ A number of recent reviews have now confirmed and expanded on these findings.^28, 29^

## Maternal health, prematurity and low birth weight are linked to increased risk of ASD

Obesity during gestation has been strongly associated with prematurity and low birth weight.^30, 31^ Obesity is considered as an inflammatory state^32^ and has been associated with activation of MCs.^33, 34^ Moreover, MCs secrete leptin^35^ and its deficiency switches MC to an anti-inflammatory phenotype.^36^ Leptin is increased both in obesity^37^ and ASD.^38^ Premature births account for about 15% of all births in the USA and premature infants (32–36 weeks) make up most of the increased rate of prematurity.^39^ Such infants are at risk for neurologic injury^40, 41^ associated with decreased attention, increased anxiety, as well as social interaction and learning difficulties.^42^

A retrospective study reported that children <33 weeks gestation were associated with a twofold higher risk of ASD.^43^ One prospective study found that 26% very low birth weight (<1500 g) infants ((*n*=91), mean age of 22 months) developed ASD.^44^ There was a higher risk of infantile autism among children with low birth weight especially in mothers >35 years, foreign born and those who had psychoactive medicines during pregnancy.^45^ Another case-control population-based cohort study among Swedish children *(n*=408, born 1974–1993), reported that the risk of ASD was associated with being small for gestational age, daily maternal smoking in early pregnancy, maternal birth outside Europe and North America, a 5-min APGAR score <7 and congenital malformations.^46^

Perinatal stress has been linked to increased risk of ASD.^18, 47^ Such stress may be linked to sexual abuse that has been associated with higher risk of ASD.^48, 49^ ASD patients are prone to stress^50^ and a meta-analysis showed a strong correlation between the presence of anxiety disorders and ASD.^51^ In fact, anxiety was significantly correlated with repetitive behaviors in children with ASD.^52^ We reported that the peptides NT^53^ and CRH^9^ secreted under stress were increased in the serum of young children with ASD, as compared with normal controls.^53^ The highest expression of NT receptors in the human brain is in the amygdala,^54^ hypothalamus and area of Broca,^55^ which regulate emotions and language, respectively. Stress can activate MCs through CRH leading to increased BBB permeability.^56^ Moreover, CRH has synergistic actions with NT, stimulating secretion of VEGF and increasing vascular permeability.^53^ Human MCs express CRHR-1,^57^ activation of which by CRH leads to VEGF secretion and BBB disruption^58^ and NT stimulates secretion of VEGF.^57^

A recent review concluded that stress during gestation increases the risk for developing atopic diseases in infants.^59^ Moreover, stress has been associated with precipitating or worsening asthma^60^ and multiple sclerosis.^61^

## Atopic diseases are strongly correlated with increased risk of ASD

Recent studies have shown strong associations between allergies, asthma, autoimmune diseases and psoriasis in the mother with increased risk for ASD in their children.^62, 63, 64^ Moreover, mothers with mastocytosis or MC activation syndrome were much more likely to have children who developed ASD.^65^

Allergies^66^ and auto-immune diseases^67, 68^ have been increasing significantly. Early reports indicated more frequent allergies in ASD children,^69, 70^ with food allergies being the most prevalent complaint, often in the absence of elevated serum IgE or positive skin tests.^71, 72, 73^ A large epidemiological study of noninstitutionalized children (*n*=92 642; 0–17 years old) showed that eczema was strongly associated with ASD and attention deficit hyperactivity disorder.^74^ Another study of atopic subjects (*n*=14 812; 3 years old) and non-atopic subjects (*n*=6944) also showed a strong association between atopy and risk of both ASD and attention deficit hyperactivity disorder.^75^ A case control study of children and young patients with ASD (*n*=5565) and controls (*n*=27 825) matched to birth year (1980–2003) and sex reported that allergies, asthma and autoimmune disorders were diagnosed more frequently, with psoriasis occurring more than twice as often, in ASD patients than controls.^76^ An experimental study actually reported neurochemical changes and autistic-like behavior in a mouse model of food allergy.^77^

MCs can be activated by fungi,^78^ such as *Aspergillus fumigatus* which triggers IgE-independent MC degranulation^79^ and fungal zymosan induces leukotriene production from human MCs.^80^ Moreover, MCs can be stimulated by aluminum and mercury.^81, 82^

## Perinatal epigenetic environmental triggers contribute to inflammation of the brain and increase risk of ASD

Environmental triggers have been increasingly invoked in ASD.^17, 19, 83, 84, 85, 86^ Chemical intolerant mothers were three times more likely to have a child who developed ASD and these children were more prone to allergies and sensitivities, including odors.^87^ Exposure to mold has been linked to decreased cognitive function in children^88^ and volatile mycotoxins have been reported to induce neuropsychiatric symptoms.^89^

Both mercury^90^ and aluminum^91, 92^ have been associated with symptom severity in children with ASD and both can stimulate MCs.^81^ Aluminum has replaced mercury as an adjuvant in vaccines, but aluminum can cause DNA damage^93^ and induce microglia TNF release.^94^ The adjuvant activity of aluminum was shown to be mediated through DNA released from dying cells, possibly through production of IgE and IgG_1_, known MC triggers.^95^ Such ‘damage-associated molecular patterns' can act as ‘alarmins'^96^ and cause inflammatory responses through toll-like receptors, which participate in immunity against bacterial infections^97, 98^ and are also expressed on MCs.^99^

Stimulated human MCs can secrete mitochondrial DNA (mtDNA) and ATP extracellularly without cell death.^100^ These mitochondrial components augmented allergic responses^101^ and could act as ‘innate pathogens' triggering inflammation and potentially contributing to ASD.^102^ mtDNA is also directly neurotoxic in rat brain slices.^103^ We reported that serum mtDNA is elevated in young autistic children as compared with controls.^104^ The pathological importance of extracellular mtDNA could be even more relevant in the subgroup of ASD patients with mitochondrial dysfunction.^105^

MCs are therefore considered important for inflammation.^23^^,^^106^

## Evidence for inflammation of the brain in ASD patients

Increasing evidence indicates that perinatal brain inflammation,^18, 107^ may contribute to the pathogenesis of neuropsychiatric disorders,^108, 109^ including ASD.^26, 110^ It was previously reported that ASD pathogenesis involves some immune^17, 111, 112, 113^ and autoimmune^102, 114^ components. Circulating auto-antibodies directed against fetal brain proteins have been reported in mothers of children with ASD^115, 116^ and in about 37% of ASD patients,^117^ implying BBB disruption which is regulated through MCs.^56, 118^ The presence of auto-brain antibodies significantly correlated with allergic symptoms.^119^

A number of inflammatory molecules have been shown to be increased in the brain and cerebrospinal fluid of many ASD patients including IL-1β, IL-6, TNF, MCP-1 and CCL8 (IL-8) ^120, 121, 122^ (Table 3). Plasma levels of IL-1β, IL-6 and IL-8 were increased in children with ASD and correlated with regression, as well as impaired communication and aberrant behavior.^123^

Analysis of cytokines in neonatal blood showed that IL-1β and IL-4 linked to severe ASD.^124^ In a previous study by some of the same authors, these cytokines were not detected apparently due to the sensitivity of the assay used.^125^ Increased maternal serum concentrations of IFN-γ, IL-4 and IL-5 during midgestation were significantly associated with a 50% increased risk of ASD.^126^

MC-derived TNF can promote Th17-dependent neutrophil recruitment.^127^ Moreover, MC-derived IL-6 and TGFβ promote the devlopment of Th17 cells.^128^ In fact, MCs can also secrete IL-17^129^ and IL-17 was reported to be increased in the serum of children with ASD.^130^ There was an increased IL-17 production from peripheral blood immune cells following mitogen stimulation, and IL-17 was further increased in ASD children with comorbid asthma.^131^ A recent paper reported that selective elimination of Th17 cells in the maternal immune activation (MIA) mouse model prevented the development of autism-like behaivor in the offspring.^132^

The MIA model was also associated with increased serum IL-6,^133^ and the autism-like behavior was absent in IL-6^−/−^ mice.^134^ We had reported that acute stress significantly increases serum IL-6 in mice that was entirely dependent on MCs, as it was absent in MC-deficient W/W^v^ mice.^135^ In fact, human MC can undergo selective release of IL-6 without degranulation.^136^ Mastocytosis patients have increased serum IL-6 that correlates with disease activity^137, 138, 139^ and children with mastocytosis had a 10-fold higher risk of developing ASD than the general population,^65^ implying activation of MCs.^27^

MCP-1 in amniotic fluid was strongly correlated with increased risk for infantile autism^140^ and MCP-1 was also elevated in archived neonatal blood specimens.^125^ MCP-1 is chemotactic for MCs,^23^ which can secrete both pre-formed and newly synthesized TNF.^141^ TGF-beta has been reported to be low in the brains of children with ASD,^142^ a finding that may contribute to the inflammatory state since TGF-beta inhibits MCs.^143, 144^

Peripheral blood mononuclear cells from patients with ASD (*n*=23) produced twice as much TNF as those from controls (*n*=13) when stimulated even by gliadin, cow's milk protein or soy.^145^ NF-κB DNA-binding activity, involved in TNF production, was twice as much in peripheral blood from patients with ASD (*n*=67) than controls (*n*=29).^146^ Neurons, astrocytes and microglia from patients with ASD had higher expression of NF-κB p65 as compared with matched controls.^147^ Moreover, signaling through NF-κB was prominent in interacting gene networks constructed from brains of ASD patients.^148^

MCs have recently been considered important in neuroinflammation.^149^

## MC–microglia interactions in the pathogenesis of ASD

Microglia, the innate brain immune cells,^150^ are important during healthy brain development because they may ‘prune' neural circuits.^151, 152^ However, abnormal microglia activation and proliferation could lead to focal inflammation and ‘choking' of normal synaptic traffic as has been reported in brains of patients with ASD.^39, 153, 154, 155^ A recent study of the transcriptomes from 104 human brain cortical tissue samples from patients with ASD identified gene clusters associated with increased microglia activation (M2) and decreased neuronal activity.^156^ As a result, microglia are now considered an important component of the pathogenesis of ASD.^157, 158^

**Figure 1:**
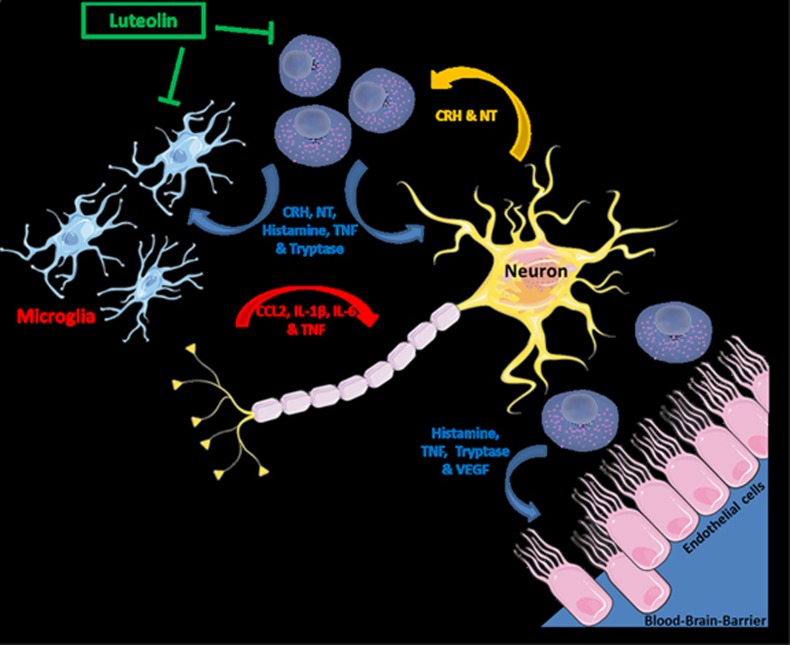
Schematic representation of the interactions among mast cells–microglia–neurons and the blood–brain barrier. Curved arrows, along with mediators associated with them, indicate action from one type of cell to another. The inhibitory action of luteolin (in box) is indicated by the inhibitory symbols (T). CRH, corticotropin-releasing hormone; IL, interleukin; NT, neurotensin; TNF, tumor necrosis factor; VEGF, vascular endothelial growth factor.

Human microglia express functional CRHR1^159^ and NTR3 (sortilin), activation of which leads to microglia proliferation.^160^ NTR3 has been implicated in neuronal viability and function^161^ and increased soluble sortilin has been associated with depression, corresponding to elevated levels of BDNF and VEGF.^162^ NT can be neurotoxic by facilitating *N*-Methyl-d-aspartate-induced excitation of cortical neurons.^163^ We recently reported that NT stimulates activation and proliferation of human microglia.^164^ We believe this is the first time that a neuropeptide elevated in ASD is shown to stimulate human microglia that are now believed to play a major role in the pathogenesis of ASD.^39, 153, 154^ NT can therefore stimulate both microglia and MCs (Figure 1).^53^

Signaling through the mammalian target of rapamycin (mTOR) has been implicated in ASD^5, 165^ and mutations of the mTOR upstream regulatory molecule phosphatase and tensin homolog (PTEN)^166^ and tuberous sclerosis complex 1 and 2 (TSC 1/2)^167^ have been associated with higher risk of ASD.^167^ We recently showed that activation of NTR3 induced activation of human cultured microglia, which was regulated by mTOR.^164^ PTEN and mTOR are also involved in MC activation and proliferation.^168^

MC-derived histamine^169^ and tryptase^170^ can stimulate microglia, findings that have led to the proposal that MC-microglia interactions are important in neuroinflammation.^171, 172^ Stimulation of brain MC in mice was recently shown to induce microglia activation and brain inflammation, inhibited by a MC stabilizer.^172^

It is, therefore, important to address neuroinflammation as a possible treatment option for ASD.

## Treatment approaches

Most children with ASD are often prescribed psychotropic medications,^173^ primarily risperidone and aripiprazole to reduce disruptive and aggressive behaviors, but these drugs have no effect on the core symptoms of ASD.^174, 175^ In fact, recent studies have questioned the benefit of psychotropic agents and have highlighted frequent adverse effects such as weight gain, sedation, tremor, movement disorders and drooling.^176^ As a result, there is increased polypharmacy^174, 177^ and risk of unwanted drug interactions.^178^

There should be concerted efforts toward developing effective treatments for ASD, such as the European Autism Interventions-A MultiCentre Study for Developing New Medications (EU-AIMS) Initiative.^179^

Immunomodulatory treatments have been considered for ASD,^180^ but few studies have been published. Some reports have hypothesized that the increase in ASD is linked to the increased use of the antipyretic acetaminophen.^181^ On the contrary, some families report that high fever reduces symptoms temporarily.^182^

### Immune Ig

Intravenous (i.v.) immunoglobulin treatment (commonly known as immune Ig) has been used in ASD.^183, 184^ In one study, i.v. Ig (200 to 400 mg kg^−1^, every 6 weeks × 2) was administered to children with ASD (*n*=10) with one child showing significant and four children showing mild improvement.^185^ Three pilot open clinical trials showed some benefit.^186, 187, 188^

The usefulness of this approach may be even more apparent in children with ASD whose plasma levels of IgG and IgM were reported to be low in spite of apparently normal numbers of B cells.^189^

### Macrophage activating factor (GcMAF)

This molecule, an endogenous glycosylated vitamin D-binding protein, which promotes macrophage cell activation, downregulated the over-activation of blood monocyte-derived macrophages observed in autistic children (*n*=22, 3–11 years old) compared with age-matched healthy developing controls (*n*=20).^190^

### Antioxidant compounds

A recent double-blind, placebo-controlled, study using the broccoli-derived anti-oxidant sulforaphane in adult patients with ASD (*n*=40, 13–27 years old, selected for their history of reduced symptoms during febrile episodes) for 18 weeks showed significant improvement (34%) in social interaction and communication using the Aberrant Behavior Checklist (ABC) scale;^191^ however, the apparent significance was due to the uncharacteristically low placebo effect (<3.3%). Placebo effects have been reported as high as 40–60% in studies of neuropsychiatric diseases.^192^

Another antioxidant, *N*-acetylcysteine (NAC), has also been tested. In one randomized, placebo-controlled, trial (*n*=13) increasing doses of NAC (900 mg per day × 4 weeks, then 900 mg twice daily × 4 weeks and finally 900 mg three times daily × 4 weeks) found no difference on the total ABC, but significant improvement on the irritability subscale.^193^ In another also randomized, double-blind, placebo-controlled, study (*n*=40), NAC added to a stable dose of risperidone, again had no effect on total ABC, but decreased the irritability subscale.^194, 195^ NAC treatment appears to be safe and well-tolerated.^195^ Similar results were obtained in a more recent randomized, double-blind, placebo-controlled clinical trial of children with ASD (*n*=40) who were given NAC (600–900 mg per day) and risperidone titrated (between 1 and 2.0 mg per day); by week 10, the NAC group showed significantly less irritability using the ABC-C irritability subscale (*P*=0.02).^196^

### Anti-inflammatory compounds

An open-label study investigated a formulation containing the natural flavonoids luteolin and quercetin ((100 mg each per softgel capsule in olive kernel oil to increase oral absorption) 1 capsule per 10 kg weight per day for 6 months) in children with ASD (4–10 years old, *n*=50) and reported significant (*P*<0.005) improvement in attention and behavior (34% in total ABC and 8.43 months in age-equivalent scores in the VABS communications domain).^197^ A subgroup of children in that study improved even more (65%) and were the ones with highest serum TNF and IL-6 at the beginning of the study, the levels of which dropped below basal levels at the end of treatment.^122^ These results indicate that objective inflammation markers may identify a subgroup of children with ASD, who are most amenable to treatment with luteolin or quercetin. A case series using the same formulation in children with ASD and atopic diseases (*n*=17, 4–12 years old) reported 65% improvement in attention and communication.^198^ Luteolin also improved ‘brain fog', characterized by reduced attention span, memory and learning^199^ in adults.

Luteolin (5, 7, 3′, 4′-tetrahydroxyflavone) is naturally found in green plants, herbs and seeds ^200^ and is structurally related to 7, 8-dihydroxyflavone, which was shown to have brain-derived neurotrophic factor (BDNF) activity^201^ (Table 3). Low BDNF was associated with autistic-like-behavior in mice^202^ and 7, 8-dihydroxyflavone reduced symptoms in a mouse model of Rett syndrome,^203^ which is strongly associated with ASD.^204^

Luteolin and its structurally related flavonol quercetin (5, 7, 11, 3′, 4′-pentahydroxyflavonol) inhibit histamine, IL-6, IL-8, TNF and tryptase release from human MCs.^205, 206^ We recently showed that tetramethoxyluteolin is a more potent inhibitor of human MCs than luteolin.^207^ Luteolin also inhibits microglial activation and proliferation,^208^ especially IL-6 release,^209^ and is neuroprotective.^210^ Luteolin also prevented autism-like behavior in a mouse ‘model' of autism.^211^ Flavonoids are generally considered safe^212, 213^ and now being increasingly discussed for the treatment of neurodegenerative disorders.^214^

## Conclusions

Substantial evidence indicates that the presence of atopic diseases increases the risk of ASD and that inflammation of the brain may be involved in the pathogenesis of ASD. Addressing allergic symptoms, as well as reducing BBB permeability and inflammation of the brain, could provide significant benefit in ASD. Luteolin analogs with better bioavailability and BDNF activity should be investigated further. Intranasal administration to penetrate the brain through the cribriform plexus could deliver anti-inflammatory molecules directly to the brain. Such formulations could further be prepared in liposomes to contain molecules that target them to microglia.

## Figures and Tables

**Table 1:** ASD comorbidities or subgroups

ADD
ADHD
Atopic diseases
Food intolerance
Gastrointestinal symptoms
Mitochondrial dysfunction
PANDAS
*PTEN* mutations
Seizures

Abbreviations: ADD, attention deficit disorder; ADHD, attention deficit hyperactivity disorder; ASD, autism spectrum disorder; PANDAS, pediatric autoimmune neuropsychiatric disorders associated with streptococcal infections; PTEN, phosphatase and tensin homolog.

**Table 2:** Perinatal conditions increasing the risk of ASD

*Strong evidence*
Allergies
Asthma
Brain autoantibodies
Brain hemorrhage
Infection
Low birth weight
Obesity
Preeclampsia
Prematurity
Psoriasis
Stress

*Moderate evidence*
Cesarean section with general anesthesia
Environmental toxin exposure
Oxytocin, prolonged use for labor induction
Psychotropic medication use
Sexual abuse

Abbreviation: ASD, autism spectrum disorder.

**Table 3:** Evidence for inflammation of the brain

*Brain*
Microglia activation
Microglia proliferation
IL-1β ↑
IL-6 ↑
IL-17 ↑
TNF ↑

*Blood*
Auto-brain antibodies ↑
IL-1β ↑
IL-6 ↑
IL-17 ↑
TNF ↑
NF-κB ↑

*Neonatal blood*
MCP-1 ↑

*Midgestational blood*
Auto-brain antibodies ↑
IL-4, IL-5, IFN-γ ↑

Abbreviations: IL, interleukin; TNF, tumor necrosis factor.
